# Architectural distortion on digital breast tomosynthesis mammograms in symptomatic breast clinics: what are the result outcomes?

**DOI:** 10.1093/bjr/tqae101

**Published:** 2024-05-14

**Authors:** Gaurav J Bansal, Riya Kale

**Affiliations:** The Breast Centre, Llandough University Hospital, Cardiff and Vale University Health Board, Honorary Teacher Cardiff University, Penarth CF64 2XX, United Kingdom; Medical student, Cardiff University, United Kingdom

**Keywords:** breast, mammogram, distortion, cancer, tomosynthesis

## Abstract

**Objectives:**

In April 2020, standard two-dimensional (2D) full-field digital mammograms were replaced with digital breast tomosynthesis (DBT) and synthesised 2D views for symptomatic breast clinics. This study aimed to evaluate the positive predictive value (PPV) for malignancy in DBT-detected Architectural distortion (AD).

**Methods:**

All mammogram reports with the word “distortion” were assessed between April 2020 and October 2022. There were 458 mammograms with the word “distortion.” After excluding mammograms with no distortion (*n* = 128), post-surgical distortion (*n* = 173), distortion with mass (*n* = 33), and unchanged distortion (*n* = 14), there were 111 patients with pure distortion. Correlation with histopathology was obtained where possible. All patients were followed for a minimum of 2 years.

**Results:**

Forty-two out of 111 patients (37.84%) with AD had a normal ultrasound (US) and were discharged. Fifty-five (49.5%) patients had sonographic correlation corresponding to the distortion, leading to US-guided biopsy. Thirteen (23.6%) had tomosynthesis-guided biopsy, and one had a skin biopsy. The PPV for malignancy was 42.34%. Malignancy diagnoses were higher with US-guided biopsies than tomosynthesis-guided biopsies, 78.1% and 30%, respectively.

**Conclusion:**

With a total malignancy rate of 42.34%, DBT-detected AD has a high enough PPV for malignancy to justify selective tissue sampling if a sonographic correlate is present or with suspicious mammograms. The chances of malignancy are higher when a sonographic correlate corresponding to AD is present.

**Advances in knowledge:**

AD on DBT/synthesized mammograms views in symptomatic breast clinic patients justifies selective sampling.

## Introduction

Architectural distortion (AD) detected on mammograms is defined as “thin straight lines radiating from a point, with focal distortion or straightening at the anterior or posterior edge of the breast parenchyma”. It may or may not be associated with a mass/density/asymmetry.^1^ Our centre replaced standard two-dimensional (2D) full-field digital mammograms (FFDM) with digital breast tomosynthesis (DBT) and 2D synthesized mammograms (SM). This change applied to all patients over 40 attending symptomatic breast clinics from April 2020. In addition to equivalent diagnostic performance to FFDM/DBT, SM/DBT leads to faster acquisition and reduces the radiation dose to approximately half, to a level comparable to FFDM alone.^2^^,^^3^

DBT reduces overlapping structures’ superimposition and summation shadow, making isolated AD easily detectable.^4^ Many studies have shown that AD incidence increases following the adoption of DBT, with some studies^5^ reporting a 2-fold increase in DBT-detected AD.

The underlying pathology of DBT-detected AD could be manifold, including malignant causes, high-risk lesions (B3), and benign causes. Previous studies have reported the positive predictive value (PPV) for malignancy of DBT-detected AD to be variable from 6.8% to 50.7%.^6–9^ This value is somewhat lower than the PPV for malignancy on standard 2D views detected AD of 43.4% to 73.6%.^6–9^ With the recent change in practice at our centre, all patients over 40 received obligatory DBT and synthesized 2D SM views. We sensed an increase in the incidence of AD following this change in practice. It became prudent to evaluate the clinical significance of DBT/SM-detected isolated AD.

This study aimed to evaluate the PPV for malignancy in DBT/SM-detected AD and summarizes its clinical management and pathological outcomes when available.

## Methods

All mammogram reports with the word “distortion” were assessed between April 2020 and October 2022 using the Hospital Picture Archiving and Communication Systems. There were a total of 458 mammograms with the word “distortion.” This was an observational cohort study of already collected data, which was anonymized for analysis. The study was performed as a departmental service evaluation project. In accordance with the hospital research departmental guidelines, formal ethical approval was not required for this observational study of precollected images. Patient consent was not deemed necessary as per local guidelines.

After excluding mammograms with no distortion (*n* = 128), post-surgical distortion (*n* = 173), distortion with mass (*n* = 33), and unchanged distortion (*n* = 14), 111 patients with isolated new distortion were included in this study.

All patients over 40 had DBT and SM views of both breasts. The complete series included DBT craniocaudal (CC) and mediolateral oblique (MLO) views of both breasts, followed by SM. We used Hologic Selenia Dimensions (Bedford, MA, United States) to obtain mammograms with improved synthesised imaging processing software (C view). Hologic Selenia is one of the three vendors along with GE, GE Healthcare Technologies Inc, Chicago, Illinois, USA and Siemens, Munich, Germany approved for SM in Europe and the United States.^10^

In this study, we evaluated isolated AD without associated mass. With the change in the practice, discriminating associated mass from AD on DBT views was more straightforward. In the study group, we included patients whose mammographic distortion was associated with microcalcification. Mammographic findings were assessed in association with clinical history in the breast clinics by experienced radiologists with more than 10 years of experience reporting mammograms and DBT views.

Percutaneous image-guided biopsy was performed in patients using the modality on which it is best visualised. Ultrasound (US) was the next step for all patients, followed by a US-guided biopsy if a sonographic correlate was present. The initial biopsy with either the US or tomosynthesis-guided approach was a 14 or 16-G needle core biopsy. Patients had DBT or US-guided vacuum-assisted biopsy (VAB) only if the initial core biopsy result was B3 and the patient was not for a diagnostic surgical excision. VAB was performed with a 10G needle to obtain adequate tissue for the diagnosis. Due to the irregular margins of AD, vacuum-assisted excision (VAE) was not attempted. This approach was part of our routine patient management pathway after a Multidisciplinary team discussion.

High-end Ultrasound machines (Canon Aplio i700 and i800, Canon Medical System USA, Inc. Tustin, California, USA) with an 8-15 Mz linear transducer in a free-hand technique were used. A marker clip was placed in the biopsied area after the US, followed by post-clip mammograms to ensure a mammographic-sonographic correlation of the US finding with AD on DBT. If the AD was best visualised on DBT and there was no definite US correlation, a DBT-guided biopsy was performed, followed by marker clip insertion. All patients with a Royal College of Radiologist Breast imaging score^11^^,^^12^ of 3 (probably benign), 4 (suspicious for malignancy) or 5 (highly suspicious of malignancy) on at least one imaging modality were assessed further. Few patients with extremely low clinical and mammographic suspicion were discharged without biopsy if no sonographic correlate was found corresponding to the mammographic distortion (M3 and U1). This approach was part of the normal departmental protocol. This “no biopsy” group had a normal clinical examination, mammograms of extremely low imaging suspicion (M3) and normal or benign US of the area of distortion.

MRI was performed in a few patients following a discussion at a multidisciplinary meeting. The following criteria were followed for performing an MRI of the breasts, although not strictly adhered to as sometimes the surgeons’ discretion was upheld. MRI was performed for problem-solving in patients with high mammographic suspicion (M4, M5) and with no US correlate and if a mammographic-guided biopsy was technically impossible or difficult. MRI was also performed on some women with dense breasts, even with low clinical and mammographic suspicion (M3). MRI was also performed in certain proven cancers for accurate sizing or as part of the Neoadjuvant chemotherapy pathway.

**Figure 1.**
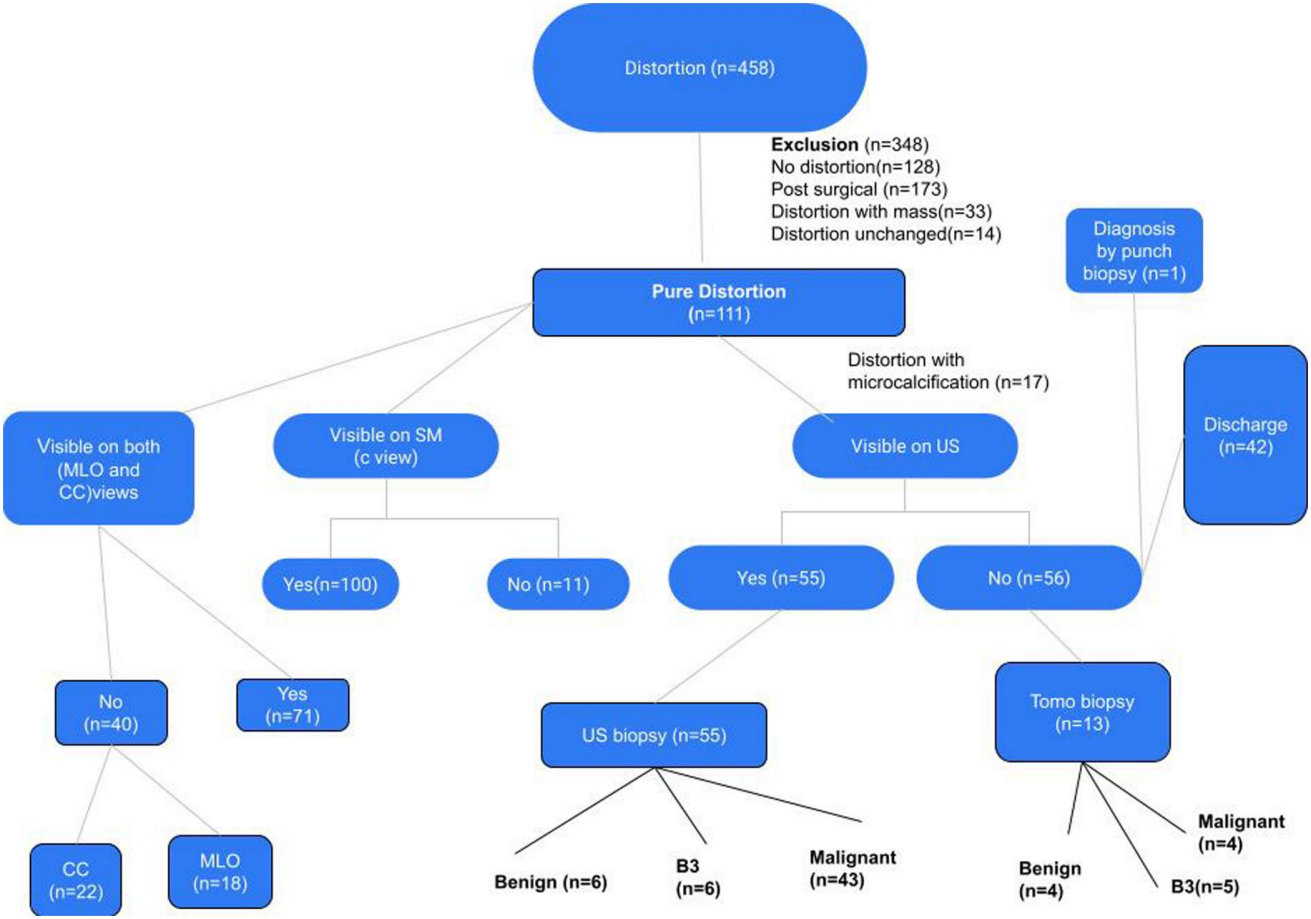
Flowchart depicting patients' pathways in the study.

A flow chart (Figure 1) depicting patients’ journeys and the management pathway was created. All patients were followed for at least 2 years, with the follow-up interval ranging from 24 to 36 months. The median age of the patients was 53 years (range 35-82 years).

## Results

In this study sample, 82% of patients had symptoms ipsilateral to the side of distortion, with lump being the most common, followed by ipsilateral nipple changes, pain, and skin dimpling. Eighteen percent of patients had contralateral symptoms, with a lump again being the most common presenting symptom. Three of these patients had bilateral symptoms.

Forty-two out of 111 patients with AD (37.84%) had a normal US or benign findings and were discharged. US was normal (U1) in all but three cases who had ductal ectasia, known granulomatous mastitis and scarring from previous surgery. These were reported as U2 and discharged. All these patients had M3 (probably benign) mammograms with low clinical suspicion. Fifty-five (49.5%) had a sonographic correlate corresponding to the distortion, leading to an US-guided biopsy, and 13 (23.6%) had a tomosynthesis-guided biopsy. One diagnosis was made by skin punch biopsy. The overall PPV for malignancy was 42.34%. Malignancy diagnoses were higher with US-guided biopsies than tomosynthesis-guided biopsies, 78.1% and 30%, respectively (see Flowchart—Figure 1).

**Figure 2.**
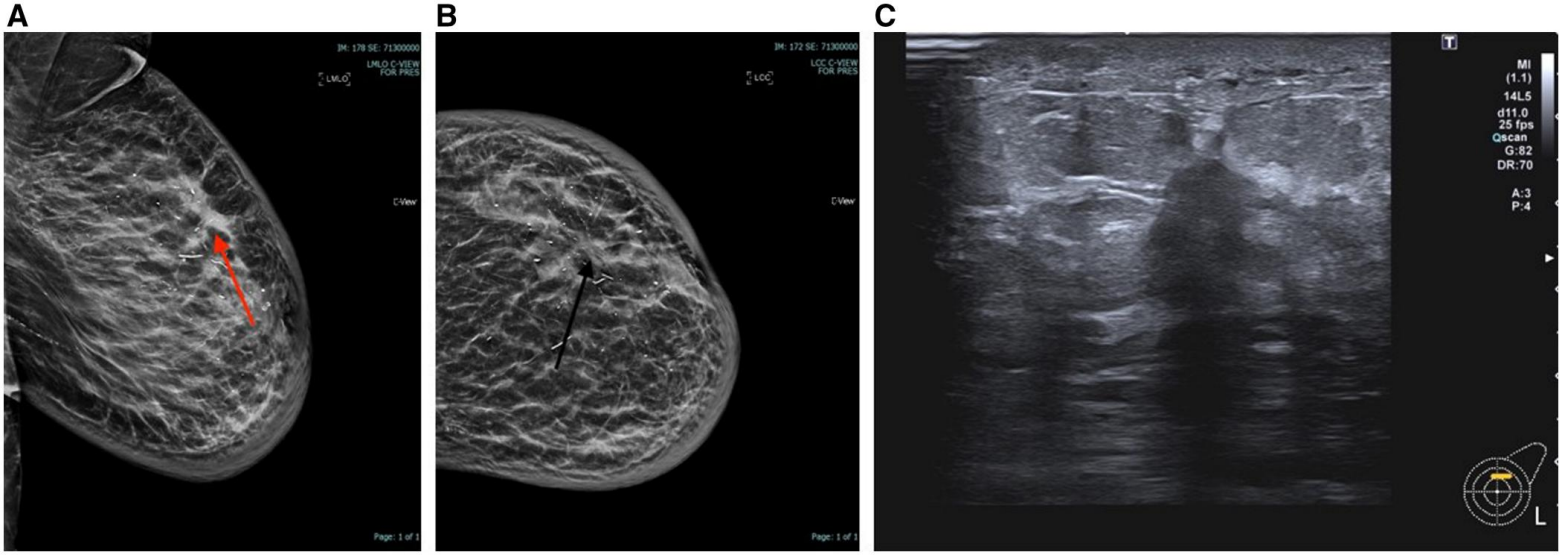
(A) MLO View showing distortion, (B) CC view showing distortion, (C) US image showing sonographic correlate corresponding to distortion. US-guided biopsy with malignant results (IDC grade 2).

**Figure 3.**
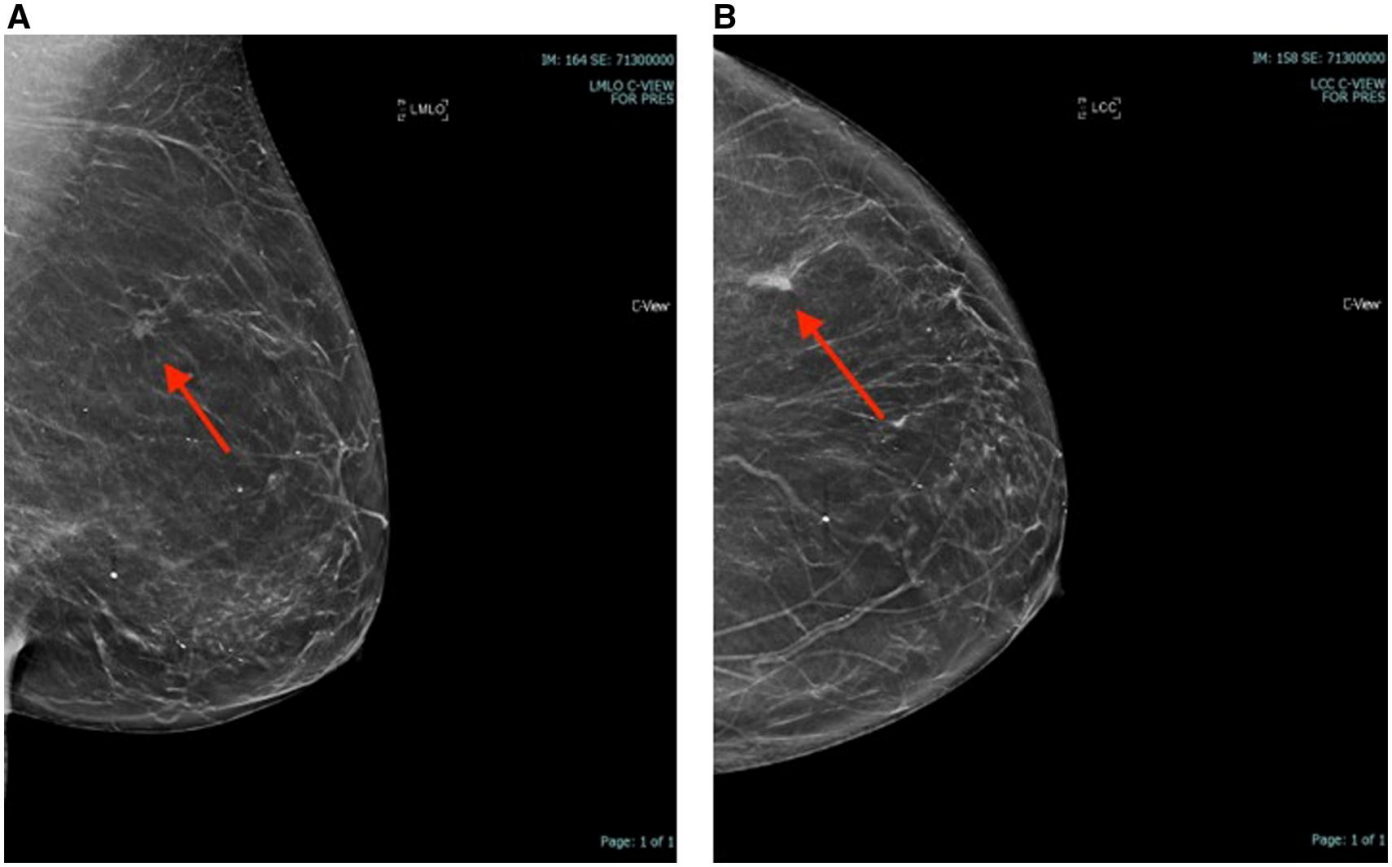
(A, B) MLO and CC views showing distortion. Tomosynthesis-guided biopsy with malignant results (IDC grade 2).

Invasive ductal cancer (IDC) was the most common histological diagnosis (*n* = 30, 62.5%), followed by Invasive Lobular Cancer (ILC) (*n* = 9, 18.7%), ductal cancer *in situ* (DCIS) (*n* = 3, 6.25%) and mixed cancers (*n* = 4, 8.33%). We found 70% (*n* = 34) of cancers to be histopathological grade 1 or 2, oestrogen receptor (ER) positive, and human epidermal growth factor receptor (HER-2) negative. HER-2 positive breast cancers and triple negative made up 5% (*n* = 3) and 10% (*n* = 6) of the total numbers, respectively. Figures 2 and 3 depict US and tomosynthesis-guided biopsy-proven malignancies, respectively.

**Figure 4.**
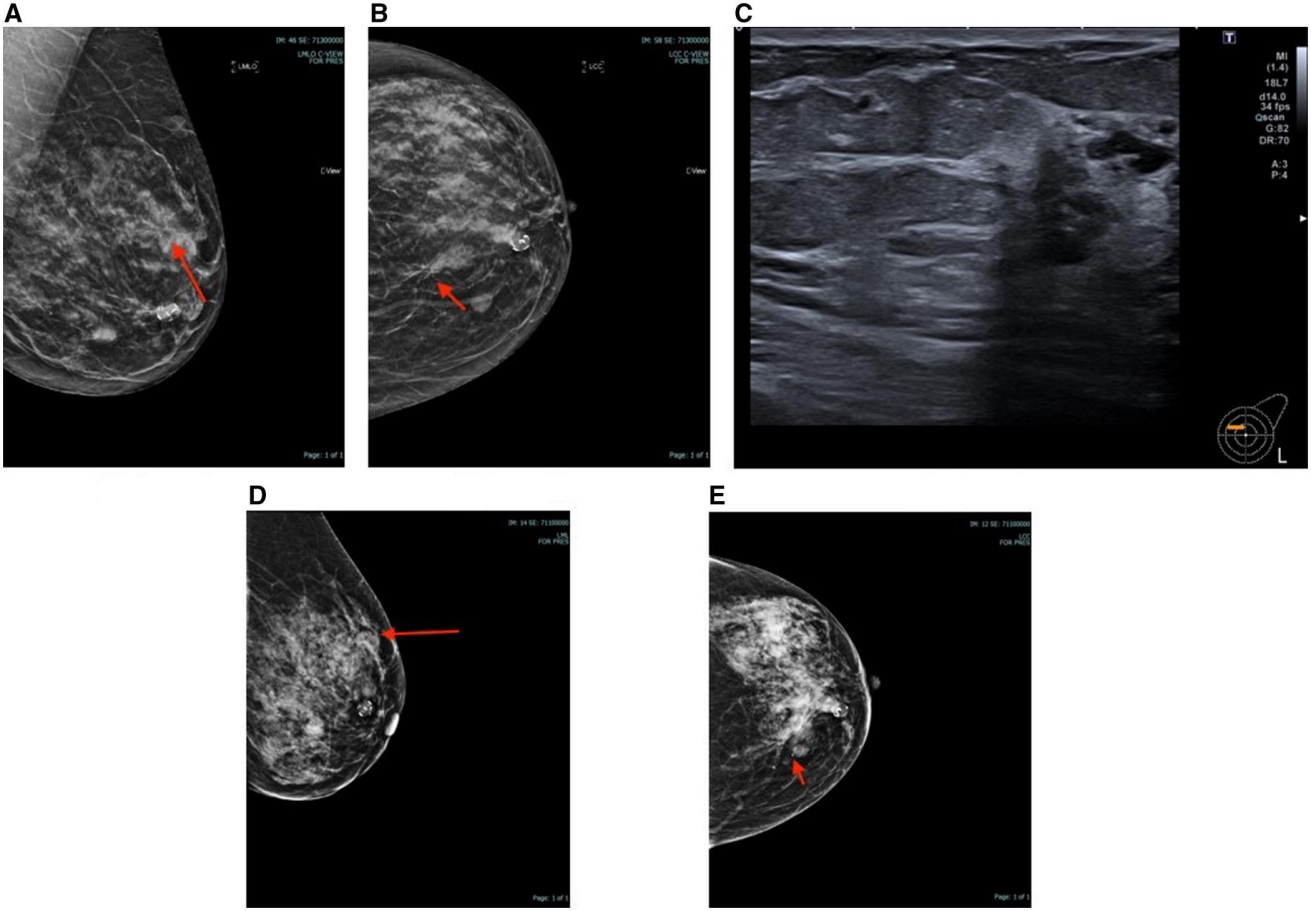
(A-C) MLO, CC, and US image corresponding to distortion. (D, E) Post clip insertion mammograms. Post-biopsy B3 (ADH) was upgraded to IDC after diagnostic excision.

**Figure 5.**
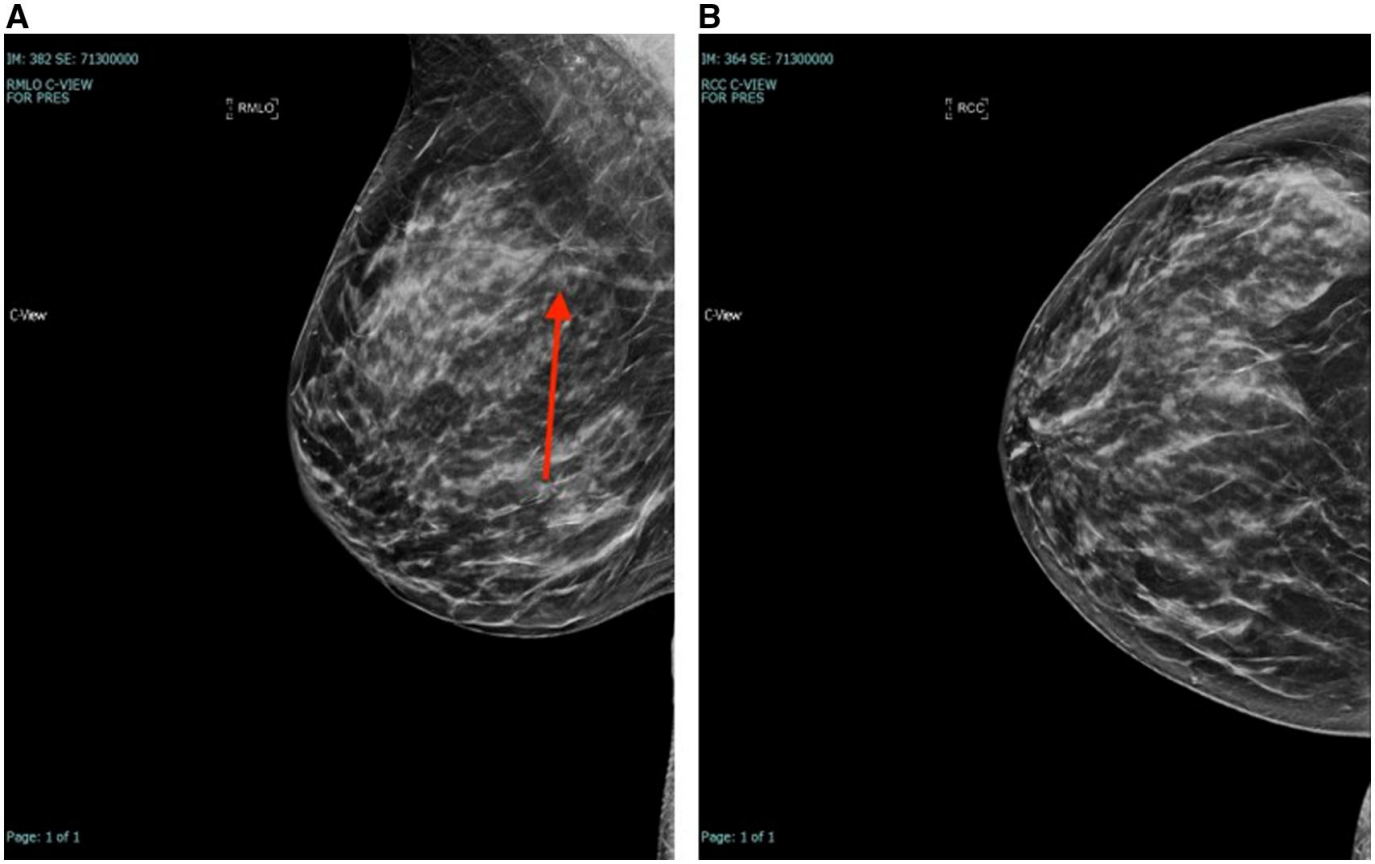
(A, B) MLO and CC views with distortion (radial scar after Tomo-guided biopsy), on enhanced surveillance.

A total B3 (high-risk lesions) rate of 9.9% (*n* = 11) was found (38% and 10.9% with tomosynthesis-guided and US-guided biopsies, respectively). Seven of the 11 B3 diagnosed were radial scars with or without atypia, three were Atypical Ductal Hyperplasia (ADH), and one was a mixture of ADH and Lobular Cancer *In Situ* (LCIS). Four of 11 were upgraded to low-grade DCIS following surgery, out of which one had VAB before surgery. One of the 11 cases was upgraded to invasive cancer following surgery. The total upgrade rate of B3 lesions was 45% in this study. Six stayed B3, two on an enhanced B3 surveillance pathway, (Figures 4 and 5)

Out of 17 patients with AD on mammograms with associated microcalcification, 10 out of 17 (58.8%) were later found to be malignant.

In our study, 43 (38.7%) patients had AD, which was only visible on one view (MLO or CC). Fourteen cases of 48 proven cancers (29.1%) were only visible as AD on one (MLO or CC) of the DBT view. Eleven of these 14 cancers were visible on CC views and three on MLO.

Forty-six of 48 proven cancers were visible on one or both views of SM. Two cancers (4%) were only visible on DBT views and not seen on SM.

In our study, MRI was performed in 6 (5.4%) cases, four of which were normal, and two had non-specific enhancements corresponding to B3 results. In one of these patients, a tomosynthesis-guided biopsy could not be performed. Patients with normal MRI results were discharged. MRI was also performed on 14 (12.6%) patients with a malignant diagnosis for accurate sizing of cancer or as part of neoadjuvant chemotherapy protocol.

All patients were followed up for a minimum of 24 months to a maximum of 36 months. None presented with cancer during this period. Twenty patients out of the 42 discharged had a mammogram at an interval of 2 weeks to 2 years from the initial mammogram, with either no change in the area of distortion or resolution of the distorted area.

## Discussion

The number of symptomatic Breast centres that have omitted standard FFDM and adopted SM/DBT imaging as their standard approach is unknown. There had been initial concerns about image quality and microcalcification characterisation on SM views. However, SM image quality has recently improved with improved image synthesising software.^10^

In a large screening study involving 224 patients,^13^ directly comparing SM with FFDM, authors found that although most cancers were equal or more conspicuous on SM when compared to FFDM, certain features like calcifications, distortion, and asymmetries <2 cm were less visible on SM than on FFDM. The same authors found distortion >2 cm was more conspicuous on SM. Similar results were found in the TOMMY trial,^14^ a large screening study, which found microcalcification <2 cm less detectable on SM+ DBT than FFDM+DBT. This threshold size difference detection between the two modalities (SM and FFDM) contrasts with the findings of Mariscotti et al,^15^ who did not see any difference in detecting various lesion morphologies by size between the two modalities. Their study included a mixture of screening and symptomatic cases. Our study did not directly compare SM with FFDM, as doing both modalities in a single patient would have doubled the radiation dose. It also differs from the above studies as it only included symptomatic patients.

In our study, 43 (38.7%) patients had AD, which was only visible on one view (MLO or CC). Previous studies^16^^,^^17^ have demonstrated that AD visible on one view should be addressed and deserves a complete diagnostic evaluation. The extra mammographic views could include spot compression views on DBT and additional full-field views at various angles. In this study, none of the patients had spot compression views and proceeded straight to the focussed US of the mammographic area of concern. Previous studies^6^^,^^16^ have cautioned against over-reliance on spot compression views, which can sometimes give a false reassurance of the absence of distortion. In this study, 29.1% of cancers were only visible in one view, with most of these visible in the CC view. Other studies have shown that one-view malignancies were more likely to be visible on CC view, and a one-view AD had a malignancy rate of 23%.^18^^,^^19^ In addition, in this study, whilst most cancers were visible on SM, two were only seen on DBT views, underscoring the importance of viewing SM in conjunction with DBT views. This finding resembles another study_,_ where 13.5% of cancers were only visible on DBT views.

In this study, we evaluated isolated AD without associated mass. Previous studies have shown a higher risk of malignancy in patients in which AD is related to a mass.^20^ Small masses can be obscured on 2D views if no tomosynthetic views are acquired. With the new practice of obtaining DBT/SM views, discriminating associated mass from AD on 3D/DBT views was more straightforward. We included patients of AD with microcalcifications and found a significant association (58.8%) with cancer when AD is associated with microcalcifications.

We identified an US correlate in 50% of cases of AD on DBT/SM. Previous studies have demonstrated that a sonographic correlate was identified in 13%-65% of cases of AD on DBT.^21^ Following a US-guided biopsy, there is a higher chance of malignancy with PPV ranging from 12.1% to 71.4% vs PPV for malignancy without a sonographic correlate (7.7%-33.3%).^9^^,^^16^^,^^20^^,^^21^ In our study, the total PPV for malignancy in all cases of AD was 42.34%. Malignancy diagnoses were higher with US-guided biopsies than with tomosynthesis-guided biopsies, 78.1% and 30%, respectively, in keeping with previous studies.^9^^,^^16^^,^^20^^,^^21^

Our findings are similar to those of another study,^22^ which involved 123 screening cases. Their case selection included biopsy-proven patients with distortion. They had a 28.8% overall PPV of malignancy, higher (66.7%) with US-guided biopsies than tomosynthesis-guided biopsies (28.2%), with distortion visible on both DBT and SM. Interestingly, the authors found an even lower PPV malignancy of 19.1% for DBT-only visible AD. Our study differs from theirs as their initial case mix had all patients with biopsy-proven AD. They also had an extremely low rate of US-correlated visible lesions and, consequently, of US-guided biopsies of 9.7%.

MRI can be a valuable tool for problem-solving due to its high negative predictive value of 98%.^21^ It is helpful in patients with questionable AD on DBT and no US correlate.^21^ In some patients, MRI would be useful if DBT-guided biopsy was technically not possible due to the location of abnormality or non-visualisation of the anomaly on biopsy images. Previous studies have shown MRI correlation for DBT-detected AD to vary from 18% to 63%^23–26^ and a malignancy rate of 17%-36%,^23–26^ warranting MR-guided biopsy. In our study, MRI was performed in 6 (5.4%) cases following equivocal AD on DBT, four of which were normal, and two had non-specific enhancements corresponding to B3 results. Patients with normal MRI results after equivocal AD were reassured and discharged. We performed MRI in a small proportion of patients. Cautious use of MRI is in keeping with recommendations of performing an MRI of breasts only in rare instances when traditional methods do not answer the questions and in conjunction with the level of overall suspicion. This cautious use of MRI is due to both a false negative rate of 2% (non-enhancing small cancers or inconspicuous cancers not seen on a background of surrounding parenchymal enhancements) and non-specific enhancement in other areas of breasts leading to false positives and further unnecessary biopsies.

Invasive ductal cancer remained the most common diagnosis on tissue diagnosis (62%), followed by ILC (18%) and a small percentage of DCIS (8%) in this study. This finding is in keeping with previous studies, where IDC and ILC accounted for 50%-71.2% and 15.4%-34.4%^27^ of all cancers presenting as AD on DBT. Some studies have found that DBT detects cancers that are smaller in size, lower in grade, and specific subtypes (ER/PR positive and HER2 negative). This finding is in keeping with our study, where we found 70% of cancers to be histopathological grade 1 or 2, ER-positive and HER2-negative. Recent studies have shown that the incidence of radial scars and other high-risk lesions like ADH, ALH, Flat epithelial atypia, papilloma (with or without atypia) and LCIS has increased following DBT and DBT-guided biopsies.^28^ A total B3 rate of 9.9% was found in this study (38% and 10.9% with tomosynthesis-guided and US-guided biopsies, respectively). 4 of 11 were upgraded to low-grade DCIS and one to invasive cancer following surgery, with a total upgrade rate of 45% in this study. These findings are similar to those of Partyka et al^16^ and Alshafeiy et al^29^ in which two cases (66%) of three were upgraded. In another study by Patel et al,^30^ none of the two B3 cases were upgraded. Most of these studies are limited by small numbers.

One of the advantages of this study is that it focuses on symptomatic breast patients with pure distortion and distortion with microcalcification only. Ours was one of the few symptomatic breast centres that omitted standard FFDM views; therefore, the findings could have important implications. Second, after introducing this new change, we followed the patients for at least 2 years to ensure no false negatives. Moreover, all readers were experienced readers of over 10 years.

One of the limitations of this study is that the actual increase in the incidence of AD relative to other mammographic findings after the adoption of DBT/SM could not be assessed. This study focused on AD after its detection and its management.

Second, there is a possibility that a small proportion of subtle distortions was not reported and, therefore, was not included in the study. This is due to inherent subjectivity in picking subtle distortions on mammograms. The authors feel these numbers would be small and will not have changed the overall results. The reason behind this is most subtle distortions would be scored as M3 and would have had an US as a minimum or an MRI in some instances.

Third, this study is small and retrospective. Our sample size was limited due to the low incidence of mammographic AD. More information could be obtained, and the findings of this study could be confirmed with a larger multicentre study. Fourthly, not all patients had DBT or US-guided vacuum biopsy. Patients had DBT or US-guided VAB only if the initial core biopsy result was B3 and the patient was not due to have a diagnostic surgical excision. This approach was part of our routine patient management pathway.

## Conclusion

Architectural distortion on DBT/SM views in symptomatic breast clinic patients justifies selective sampling. There are more chances of malignancy in AD associated with microcalcification and if a sonographic correlate corresponding to AD is present. The type of malignancy is mostly low-grade, ER-positive, and HER2-negative cancers. A total B3 rate of 10% following a tissue diagnosis of AD, slightly more after tomosynthesis-guided biopsies, is worth noting.
